# Comparative Genomic Analysis of *Dactylonectria torresensis* Strains from Grapevine, Soil and Weed Highlights Potential Mechanisms in Pathogenicity and Endophytic Lifestyle

**DOI:** 10.3390/jof6040255

**Published:** 2020-10-29

**Authors:** David Gramaje, Carmen Berlanas, María del Pilar Martínez-Diz, Emilia Diaz-Losada, Livio Antonielli, Sabrina Beier, Markus Gorfer, Monika Schmoll, Stéphane Compant

**Affiliations:** 1 Instituto de Ciencias de la Vid y del Vino (ICVV), Consejo Superior de Investigaciones Científicas, Universidad de la Rioja, Gobierno de La Rioja, Ctra. LO-20 Salida 13, 26007 Logroño, Spain; carmen.berlanas@icvv.es; 2 Estación de Viticultura e Enoloxía de Galicia (AGACAL-EVEGA), Ponte San Clodio s/n, 32427 Ourense, Spain; pilar.martinez.diz@xunta.gal (M.d.P.M.-D.); emilia.diaz.losada@xunta.gal (E.D.-L.); 3 Facultade de Ciencias, Universidade da Coruña, Zapateira, 15071 A Coruña, Spain; 4 Bioresources Unit, Center for Health & Bioresources, AIT Austrian Institute of Technology GmbH, Konrad Lorenz Straße 24, 3430 Tulln, Austria; Livio.Antonielli@ait.ac.at (L.A.); sabrina.beier@ait.ac.at (S.B.); Markus.Gorfer@ait.ac.at (M.G.); Monika.Schmoll@ait.ac.at (M.S.); Stephane.Compant@ait.ac.at (S.C.)

**Keywords:** black-foot disease, high-throughput next generation sequencing, *Vitis vinifera* L.

## Abstract

The soil-borne fungus *Dactylonectria torresensis* is the most common causal agent of black-foot disease in Europe. However, there is a lack of understanding on how this fungus can provoke plant symptoms. In this study, we sequenced, annotated and analyzed the genomes of three isolates of *D. torresensis* collected from asymptomatic vine, weed and soil. Sequenced genomes were further compared to those of 27 fungal species including root and aerial pathogens, white rot degraders, indoor biodeterioration agents, saprotrophs, dark septate endophytes and mycorrhiza. Strains of *D. torresensis* present genomes with between 64 and 65 Mbp and with up to 18,548 predicted genes for each strain. Average Nucleotide Identity (ANI) shows that strains are different according to genome contents. Clusters of orthologous groups were compared, and clusters of genes related to necroses were particularly detected in all strains of *D. torresensis* (necrosis inducing peptides and proteins, and ethylene inducing peptides) as well as several genes involved in resistance against fungicides frequently used in viticulture such as copper. Interestingly, an expanded high number of genes related to carbohydrate-active enzymes were detected in each *Dactylonectria* strain, especially those related to glycoside hydrolases that could be involved in penetration of plant tissues or pathogenicity. An increased number of candidate genes for CAZyme classes AA9 and AA3-1 supports the ability of strains to efficiently degrade plant material. High numbers of genes of *D. torresensis* related to secretome and small secreted proteins were further characterized. Moreover, the presence of several gene clusters such as fujikurin-like genes was detected and were normally found in *Fusarium* *fujikuroi*, that have been linked to fungal pathogenicity. The phenotypes of the three strains investigated showed further difference in light response. We found that *Dactylonectria* strains have an increased number of photoreceptor encoding genes and we showed sequence alterations. Altogether, the results highlight several gene clusters present in *D. torresensis* strains that could be linked to endophytic lifestyle, pathogenicity, plant maceration and degradation of plant tissues as well as adaptation to soil contaminated with metals and metalloids and light response.

## 1. Introduction

The soil-borne fungus *Dactylonectria torresensis* is the most common causal agent of black-foot disease in Europe [1,2,3], one of the most important destructive diseases in grapevine (*Vitis vinifera* L.), which has a devastating effect on grapevine production worldwide [4]. It is well known that *D. torresensis* is common in the soil and causes infection of grafted vines after some months of growth in nursery soils and in young vineyards, especially during the first five years after planting [1,2]. Young vines affected by *D. torresensis* generally appear normal at planting but differences in vigour become marked with reduced trunk growth, shortened internodes, and reduced foliage/canopy. Foliar symptoms may appear as small leaves with interveinal chlorosis, followed by necrosis and early defoliation [5]. Removal of the rootstock bark of declining plants reveals further black discolouration and necrosis of wood tissues that develop from the base of the rootstock. Below ground, symptoms include reduced total root biomass, low numbers of feeder roots, and black, sunken and necrotic root lesions [4].

Although the disease cycle of *D. torresensis* on grapevines has not been specifically studied, the behavior of *Cylindrocarpon*-like asexual morphs on other hosts [6,7] has indicated that conidia and chlamydospores are likely to be produced on the diseased roots and stem bases of infected vines. The conidia are apparently dispersed in soil water and the chlamydospores can allow the organism to survive in the soil for a number of years [8]. Previous research reports have shown that contact between these spores and the grapevine roots or callused stem bases results in high rates of infection [9,10]. Infection can occur through the small wounds made when roots on the callused cuttings break off during the planting process or through the incomplete callusing of the basal ends of the cuttings [4].

Concerning *Dactylonectria*, an alternative that has been poorly addressed is that these fungi associated with black-foot disease have a dual role: a pathogenic lifestyle on certain plants and a non-pathogenic one on others. For instance, Agustí-Brisach et al. [11] reported isolation of *Dactylonectria macrodidyma* complex, which comprehends *Dactylonectria alcacerensis*, *D. estremocensis*, *D. macrodidyma*, *D. novozelandica* and *D. torresensis*, from 26 of 52 asymptomatic weed species growing in propagation field nurseries and vineyards, with these strains being pathogenic to grapevine seedlings in potted assays. Langenhoven et al. [12] isolated several black-foot disease fungi from asymptomatic plants in South Africa, including grapevine, cereals and brasicaceous crops. Recently, Berlanas et al. [13] reported the occurrence of 13 species associated with black-foot disease from the asymptomatic inner tissues of surface sterilized secondary roots of grapevine grafted plants ready to be sold to growers in Spain. The fact that plant pathogens can be non-pathogenic endophytes on other plants has important implications, such as asymptomatic plants inadvertently serving as reservoirs of inoculum and potentially initiating epidemics in other crops [14], or even serving as sources of hidden diversity of plant-pathogenic species.

Regardless of biological, chemical, or cultural measures, no effective management strategies for *D. torresensis* are currently available to avoid fungal infection and/or to eliminate this pathogen once plants are infected [15]. Despite the importance and necessity of controlling black-foot disease, the molecular mechanisms of pathogenesis in grapevine and other secondary hosts, and the genetic basis for host specificity are still poorly understood. To date, most investigations into the nature of host-specific adaptations have focused on differences between species of plant pathogens, while fewer studies have been conducted to investigate and explain the intraspecific diversity of host-specific adaptations. In addition, no genomic and transcriptomic studies have been conducted for *D. torresensis* on grapevines although the genome sequence of its sister species *D. macrodidyma* was made public by Malapi-Wight et al. [16].

Although single genome analysis facilitates better insights into the biology of a pathogen, comparative analysis of multiple genomes can often reveal a significantly greater amount of information on the physiology and evolution of a pathogen [17]. In this study, we analyzed the genomes of three isolates of *D. torresensis* collected from asymptomatic vine and weed, and soil. The main objectives of this study were to (i) identify the genomic characteristics of these fungi, (ii) understand the genetic variation among the sequenced species, (iii) identify genes potentially involved in niche specialization within species, (iv) to identify fungal adaptations to the endophytic or pathogenic lifestyles, and (v) to identify unique and shared genes and pathways related to virulence in *D. torresensis.*

## 2. Materials and Methods

### 2.1. Fungal Strains and Culture Collection

**Figure 1:**
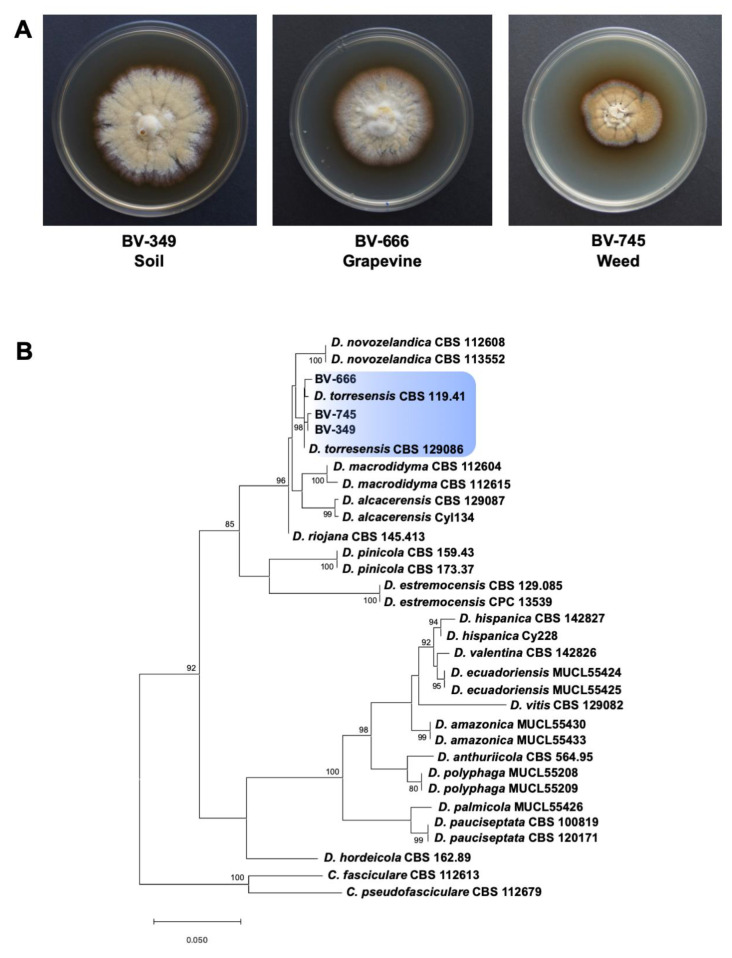
(**A**) Colony morphology of *Dactylonectria* *torresensis* strains isolated from soil, weed or grapevine. (**B**) Maximum likelihood phylogeny of *Dactylonectria* spp. as estimated from the alignment of the histone H3 gene sequences. Maximum likelihood bootstrap percentages are indicated at the nodes. Support values less than 70% bootstrap are omitted. The tree was rooted to *Campylocarpon fasciculare* (CBS 112613) and *Ca. pseudofasciculare* (CBS 112679). The scale bar indicates 0.05 expected changes per site.

Fungal strains included in the study were isolated from the weed species *Solanum nigrum* (BV-745), grapevine rootstock 110 Richter (BV-666) and soil samples (BV-349) collected in 2017 in a single grapevine nursery field in Mendavia (Navarra, Spain) (Figure 1A). Isolation from weed and grapevine were made from the asymptomatic endosphere tissue of roots. Sections of externally symptomless roots (1–2 cm long and 1–3 mm diameter) were cut, washed under running tap water, surface disinfested for 1 min in a 1.5% sodium hypochlorite solution, and washed twice with sterile distilled water. The bark was carefully peeled out and the endosphere tissue was plated onto malt extract agar (MEA; Biokar-Diagnostics, Zac de Ther, France) supplemented with streptomycin sulfate (MEAS) at 0.4 g.L^–1^ (Sigma-Aldrich, St. Louis, MO, USA). Isolation from soil samples were performed by plating them onto the Glucose-Faba Bean Rose Bengal Agar (GFBRBA) semi-selective culture medium as described by Berlanas et al. [2]. All isolates were single-spored in order to obtain pure cultures and stored in filter paper at −20 °C.

### 2.2. DNA Isolation and Fungal Identification

Fungal strains BV-349, BV-666 and BV-745 were grown on potato dextrose agar (PDA; Conda Laboratories, Madrid, Spain) plates for 7 days at room temperature. Mycelium was scraped from plates with the scalpel, transferred to the mortar and grinded with pestle in liquid nitrogen to get fine powder. One hundred mg of powder was taken for the DNA isolation using DNeasy Plant Mini Kit (Qiagen, Hilden, Germany) following manufacturer procedure as described by Berlanas et al. [13]. Obtained DNA was cleaned and concentrated using Amicon Ultra-0.5 mL Centrifugal Filters for DNA and Protein Purification and Concentration with cut off 30 kDa (Millipore-Merck, Bedford, MA, USA).

The identification of black-foot pathogens was made by sequencing part of the histone H3 gene. PCR conditions and sequence analysis were performed according to Berlanas et al. [13]. Maximum Likelihood (ML) was performed on the individual gene alignment in MEGA v. 6 [18] using the best fit model as estimated with the Bayesian information criterion in jModelTest 2.1.10 [19]. Branch support was calculated from 1000 bootstrap replicates for dataset. *Campylocarpon fasciculare* (CBS 112613) and *Ca. pseudofasciculare* (CBS 112679) were used as outgroups in the phylogenetic analysis.

### 2.3. Genome Sequencing and Assembly

Fungal genomes were sequenced with Illumina NextSeq 500 using the V2 reagent kit (2 × 150 bp) and generating a total of 50 million PE reads (LGC Genomics GmbH, Berlin). Illumina raw data were checked with FastQC [20] and possible PhiX contaminant sequences removed with Bowtie2 [21]. The presence of adapters was checked, reads were quality filtered and trimmed using Trimmomatic v.0.36 [22]. Genome assembly was carried out with SPAdes v.3.11.1 in careful mode, with k-mer size 21, 33, 55, 77, 99, 127 [23]. Contig coverage was assessed by mapping corrected reads against the assembled contigs with Bowtie 2 and BAM files were read with QualiMap v.2.2.1 [24]. Small contigs (<500 bp) with low coverage (<2×) were removed. The presence of contaminant contigs was ascertained using BlobTools v.1.0.1 [25] and the genome assembly quality of filtered contigs was evaluated with QUAST v.4.6.0 [26]. Genome completeness reconstruction was assessed with BUSCO v.3.0.2 [27]. Taxonomic affiliation was confirmed by targeting and extracting the complete Internal Transcribed Spacer (ITS) with ITSx v.1.1 [28] and locally aligning the sequence (blastn) against the UNITE web-based database (https://unite.ut.ee/) [29].

### 2.4. Average Nucleotide Identity

Previously available genome sequences of other related fungal organisms, later included in the comparative analysis, were downloaded from the NCBI repository with the ncbi-genome-download Python script [30]. Whole genome relatedness of *de-novo* assembled genomes and downloaded genomes were assessed using Average Nucleotide Identity (ANI) analysis with BLAST method [31]. The selected fungi had at least one of the following features in common with *D. torresensis*: root colonization (including pathogenic, beneficial endophytic and mycorrhizal colonization), plant pathogenesis and plant biomass degradation. These fungi were: *Ilyonectria destructans* C1, *Ilyonectria mors-panacis* g3b [32], *Dactylonectria macrodidyma* JAC15-245 [16], *Botrytis cinerea* B05.10 [33], *Fusarium graminearum* PH-1 [34], *Leptosphaeria maculans* JN3 [35], *Magnaporthe oryzae* 70-15 [36], *Serendipita indica* DSM 11,827 [37], *Ustilago bromivora* UBRO v3 [38], *Botryosphaeria dothidea* LW030101 [39], *Trichoderma reesei* QM6a [40], *Phaeomoniella chlamydospora* UCRPC4 [41], *Rhizoctonia solani* AG-3 [42], *Tulasnella calospora* AL13/4D [43], *Phaeoacremonium minimum* UCRPA7 [44], *Phaeoacremonium* sp. FL0889 (Joint Genomics Institute [JGI], http://fungalgenomes.org), *Serpula lacrymans* S7.3 [45], *Phanerochaete chrysosporium* ATCC 20,696 [46], *Rhizophagus irregularis* DAOM 181,602 [47], *Laccaria bicolor* S238N-H82 [48], *Tuber melanosporum* Mel28 [49], *Phaeomoniella chlamydospora* RR-HG1 [50], *Cadophora malorum* Mo12 [51], *Oidiodendron maius* Zn [43], *Periconia macrospinosa* DSE2036 [52], *Verticilium dahliae* Vdsl17 [53] and *Nectria haemotococca* mpVI [54].

### 2.5. Gene Prediction and Genome Annotation

The gene prediction of *de-novo* assembled genomes was conducted in Maker v.3.00.0 [55] generating a consensus prediction based on the following strategy: (1) low-complexity (simple) and interspersed (complex) repetitive elements were masked with RepeatMasker v.4.0.5; (2) *ab-initio* unsupervised gene finding was made with GeneMark-ES v.4.32 [56]; (3) a gene prediction based on precomputed *Fusarium graminearum* model was performed with Augustus v.3.2.3 [57]. The sequences of the previously available fungal genomes were either *de-novo* annotated or re-annotated, using data accessible from the JGI (https://jgi.doe.gov/). The gene prediction was executed in Maker, combining the following information: GeneMark-ES ab-initio gene prediction, available proteins from same or related organisms, existing Augustus gene prediction models from related fungal taxa.

Amino acid FASTA files were then annotated with the accelerated blastp implemented in DIAMOND v.0.9.24.125 using the NCBI fungal protein RefSeq database.

### 2.6. Functional Annotation of Predicted Genes

Functional annotation was carried out using a local installation of the whole eukaryotic orthologous eggNOG v.4.5.1 database [58]. Carbohydrate-active enzymes (CAZymes) were classified in *Dactylonectria* strains BV-349, BV-666 and BV-745 using the dbCAN Hmmer-based classification system with *E*-Value < 1 × 10^−15^, coverage > 0.35 [59] at http://bcb.unl.edu/dbCAN2/. Similar annotation was done for 27 fungal genomes including root pathogens, aerial pathogens, trunk or cane pathogens, white rot degraders, indoor biodeterioration agents, saprotrophs, dark septate endophytes and mycorrhiza. All positive hits were manually examined for final validation. All CAZyme classes, including Glycoside Hydrolases (GH), Carbohydrate Esterases (CE), Glycoside Transferases (GT), Polysaccharide Lyases (PL) and Carbohydrate-Binding Modules (CBM) were considered. Circos online software at http://mkweb.bcgsc.ca/tableviewer/was further used for data visualization and comparison of CAZymes with other fungi.

The secretome tool software http://genomics.cicbiogune.es/SECRETOOL**/** was also utilized for general secretome analysis and small secreted proteins (ssp) using the default settings for eucaryotic genomes. Comparisons were further carried out with the data from the 27 other fungal genomes.

Antismash analysis was finally performed for prediction of genes related to secondary metabolites (https://fungismash.secondarymetabolites.org/).

### 2.7. Analysis of Growth and Light Response

Strains were cultivated on malt extract (3% *w*/*v*), Mandels Andreotti minimal medium [60] or synthetic nutrient poor agar (SNA) medium [61] in daylight (12 h:12 h cycles of light and darkness) or constant darkness for 72 h for assessment of hyphal extension and light response. Fungal growth on Petri dishes were documented photographically.

## 3. Results and Discussion

### 3.1. Fungal Strains

A phylogenetic analysis was performed with *Dactylonectria* strains isolated from the weed species *Solanum nigrum* (BV-745), grapevine rootstock 110 Richter (BV-666) and soil samples (BV-349) to identify them at species-level. The Bayesian Information Criterion (BIC) best-fit nucleotide substitution model identified by jModelTest was Hasegawa-Kishino-Yano model (HKY) with gamma distributed with invariant sites rates (G + I) for the *Dactylonectria* analysis. Alignment of 31 *Dactylonectria* sequences resulted in a 540-character dataset. The three isolates clustered strongly (>98%) with the type specimens of *D. torresensis* (CBS 129,086 and CBS 119.41) (Figure 1B). Strains BV-666 and BV-745 were isolated from asymptomatic vine and weed, respectively. Recent studies have suggested that black-foot fungi have a non-pathogenic endophytic phase [12,13] and may become pathogenic to grapevine after different abiotic and/or biotic stresses conditions and thus, they are considered as latent pathogens in grapevine. Several factors have been reported to be determinants in triggering pathogenicity in an endophyte that was previously asymptomatic, such as the nutrient status, changes in plant gene expression, habitat, host genotype or the locally occurring abiotic stresses that might reduce host fitness, resulting in bias of this delicate equilibrium and thus influencing the symptom expression in plants [62]. Abiotic stress factors in new plantations and grapevine nursery fields include water stress, J-rooting, winter-kill, waterlogging, soil compaction, nutrition deficiency and/or overcropping [15].

### 3.2. Genomes

The genomes of three *D. torresensis* strains isolated from grapevine, weed or soil were sequenced and annotated. Strain BV-349 has a genome size of 64.42 Mb (GC content: 50.67%) while strain BV-666 and strain BV-745 have 65.33 Mb (GC content: 50.17%) and 64.21 Mb (GC content: 50.23%), respectively and with 19,102 predicted proteins for strain BV-349, 19,090 for strain BV-666, and 18,724 for strain BV-745 (Table 1). Single-copy ortholog analysis reported a genome assembly completeness of 98.6% for strain BV-349, 98.3% for BV-666 and 99.0% for BV-745. ANI analysis showed that strains are different at genome level (Supplementary File 1: Figure S1). The number of transposon-related proteins in the three strains is 117, 139 and 143, respectively.

### 3.3. COG Categories and Eggnog Analysis

Using COG categories and eggnog database, we analyzed each group of gene categories (Table 2) and searched which potential genes could be involved in pathogenicity or endophytism. Each strain contains genes related to ethylene induced peptide-related gene and necrosis inducing protein (NPP1) (Table 3). A high number of genes related to carbohydrate-actives enzymes were further detected in each strain. Other interesting genes to highlight were genes related to heavy metal or metalloid resistance such as copper, cadmium and others that could be detected in a vineyard (Table 3). Other genes of interest were those related to salicylate hydrolase, which degrades salicylic acid normally involved in signalling of plant defense reaction, siderophore as an iron chelator and auxin as a phytohormone (Table 3). Only a few fungal salicylate hydroxylase enzymes have been reported such as in the endophyte *Epichloë festucae* or in the pathogen *Fusarium graminearum* causing Fusarium head blight (FHB) [63]. Salicylate hydroxylase enzymatic activities have been also found in *Trichosporon cutaneum* and other *Fusarium* spp. [64,65]. Interestingly, no symptom was recorded in the weed and vine and the strains were established as non-pathogenic endophytes, suggesting the possibility that degradation of salicylic acid is a factor on how *Dactylonectria* strains avoid plant defense reaction.

### 3.4. CAZymes

A high number of genes related to carbohydrate metabolism was detected in the genomes of all three *Dactylonectria* strains investigated. Previously, CAZyme encoding genes were shown to have roles during infection of plants by fungal pathogens [39]. Therefore, we further analyzed the genomes of *Dactylonectria* strains BV-349, BV-666, BV-745 for CAZymes related genes. The strains contain 1140, 1116, and 1133 genes encoding putative CAZymes, respectively (Table 3).

**Figure 2:**
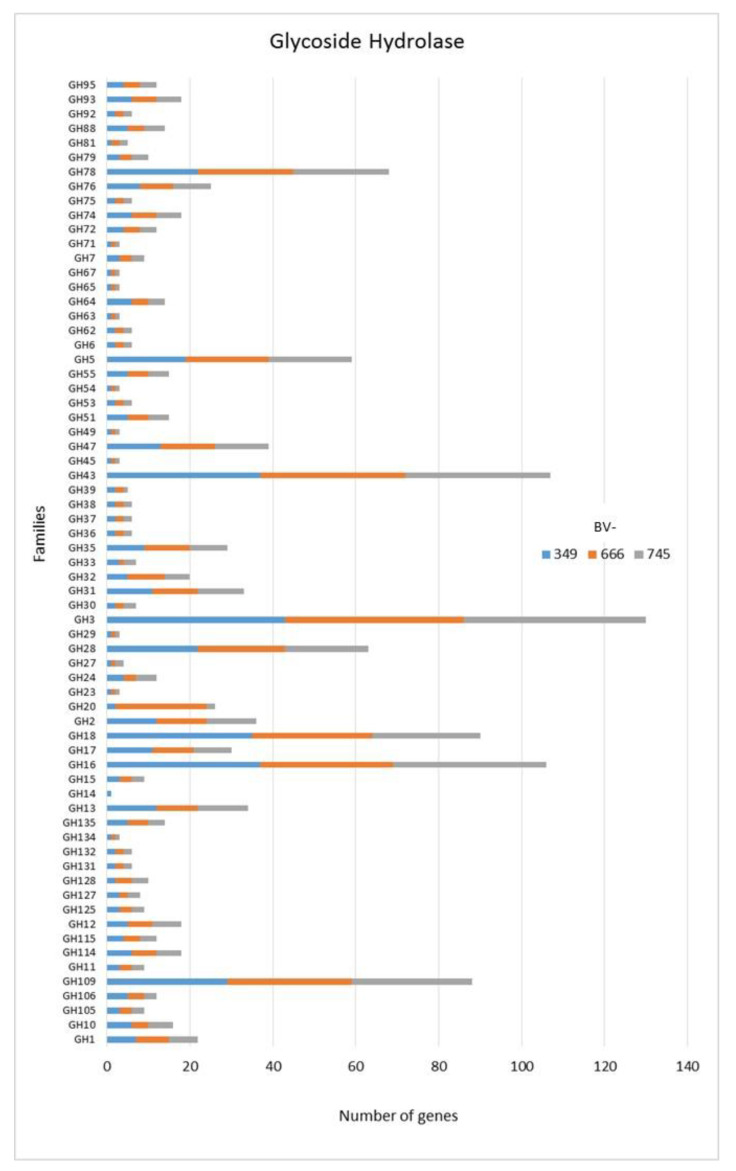
Numbers of genes related to glycoside hydrolases in *Dactylonectria* *torresensis* strains BV-349, BV-666, BV-745.

As for many fungi, among CAZymes related genes, the Glycoside Hydrolase (GH) superfamily is the most represented class in the three *Dactylonectria* strains (Figure 2). We found that some GH genes were represented by a number of genes >20 as for GH78 (Figure 2) corresponding to α-L-rhamnosidase/rhamnogalacturonan α-L-rhamnohydrolase/L-Rhap-α-1,3-D-Apif -specific α-1,3-L-rhamnosidase). Also, GH43 was over-represented. GH43 has functions like β-xylosidase/α-L-arabinofuranosidase/xylanase/α-L-arabinofuranosidase/exo-β-1,3-galactanase/β-D-galactofuranosi-dase. GH3 was also highly represented in each genome and corresponds to β-glucosidase/xylan 1,4-β-xylosidase/β-glucosylceramidase/β-N-acetylhexosaminidase/α-L-arabinofuranosidase/isoprime-verose-producing oligoxyloglucan hydrolase/coniferin β-glucosidase/exo-1,3-1,4-glucanase or β-N-acetylglucosaminide phosphorylases (Figure 2). Additionally, GH28 was highly present (Figure 2). GH28 has potential activities like polygalacturonase/α-L-rhamnosidase/rhamno-galacturonase/rhamnogalacturonan α-1,2-galacturono-hydrolase/endo-xylogalacturonan hydrolase. Lastly, GH18 (chitinase/lysozyme/endo-β-N-acetylglucosaminidase/peptidoglycan hydrolase/Nod factor hydrolase/xylanase inhibitor/concanavalin B or narbonin), GH16 (xyloglucan/xyloglucosyltransferase/β-agarase/κ-carrageenase/xyloglucanase/β-porphyranase/hya-luronidase/endo-β-1,4-galactosidase/chitin β-1,6-glucanosyltransferase), and GH109 (α-N-acetylgalactosaminidase) were also represented by a number of genes >20 (Figure 2).

**Figure 3:**
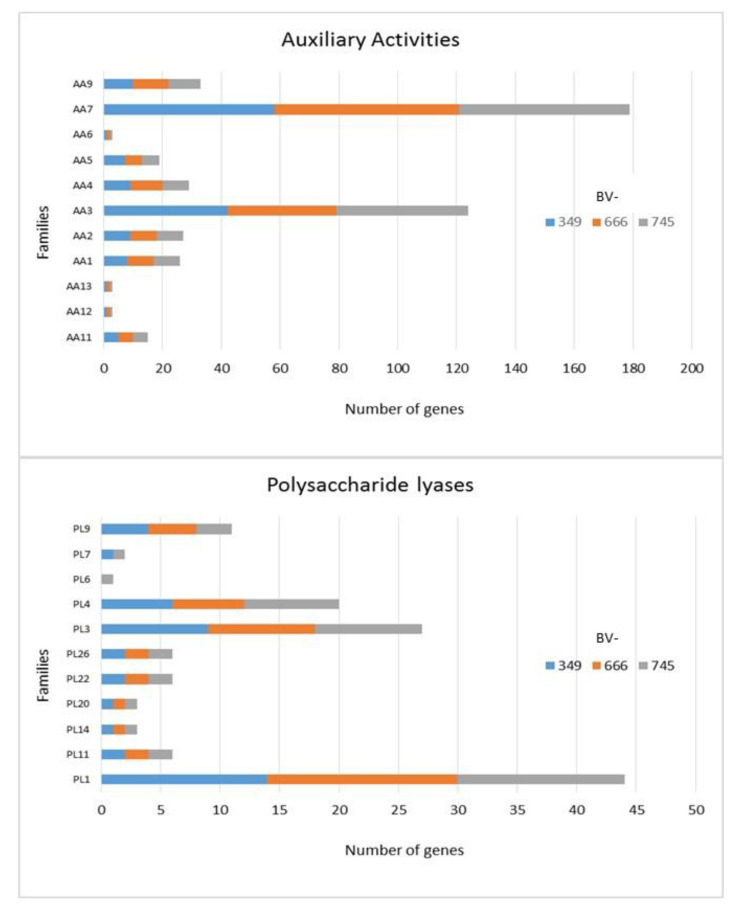
Numbers of genes related to auxiliary activities and polysaccharide lyases in *Dactylonectria* *torresensis* strains BV-349, BV-666, BV-745.

**Figure 4:**
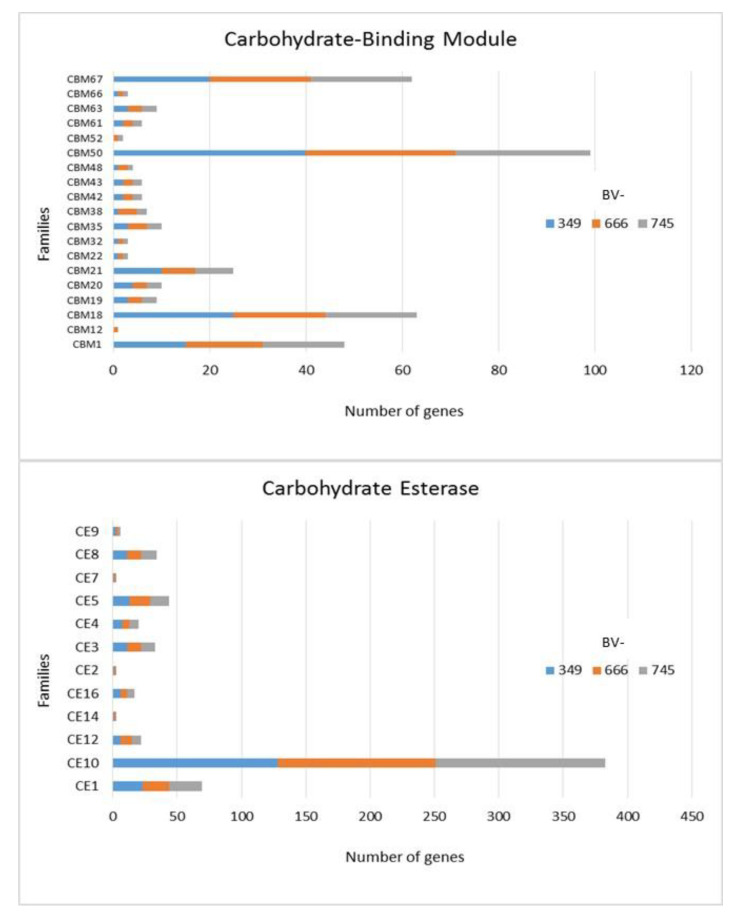
Numbers of genes related to carbohydrate-binding modules and carbohydrate esterases in *Dactylonectria* *torresensis* strains BV-349, BV-666, BV-745.

**Figure 5:**
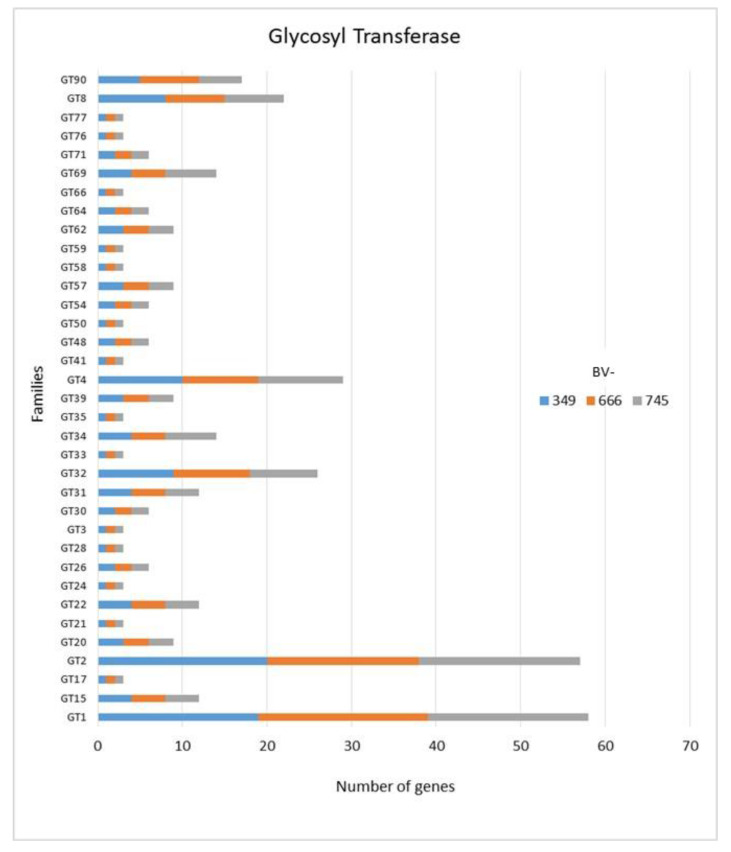
Numbers of genes related to glycosyl transferases in *Dactylonectria* *torresensis* strains BV-349, BV-666, BV-745.

In addition to GH, polysaccharide lyase related genes (PCWDE) were also detected in the three genomes of *D. torresensis* with PL1 being the most present family followed by PL3, PL4, PL9 and others (PL6, 7, 11, 14, 20, 22, 26; Figure 3). Genes related to glycosyl transferase (GT1, GT2, GT4), carbohydrate esterase (CE10), carbohydrate binding module (CBM67, CBM50) and auxiliary activities (AA7, AA3) were also further highly present in the three genomes (Figure 3, Figure 4 and Figure 5). Of those, particularly the expansion in GT1, PL1 and PL3 gene families was found to be characteristic for plant pathogenic fungi [66].

**Figure 6:**
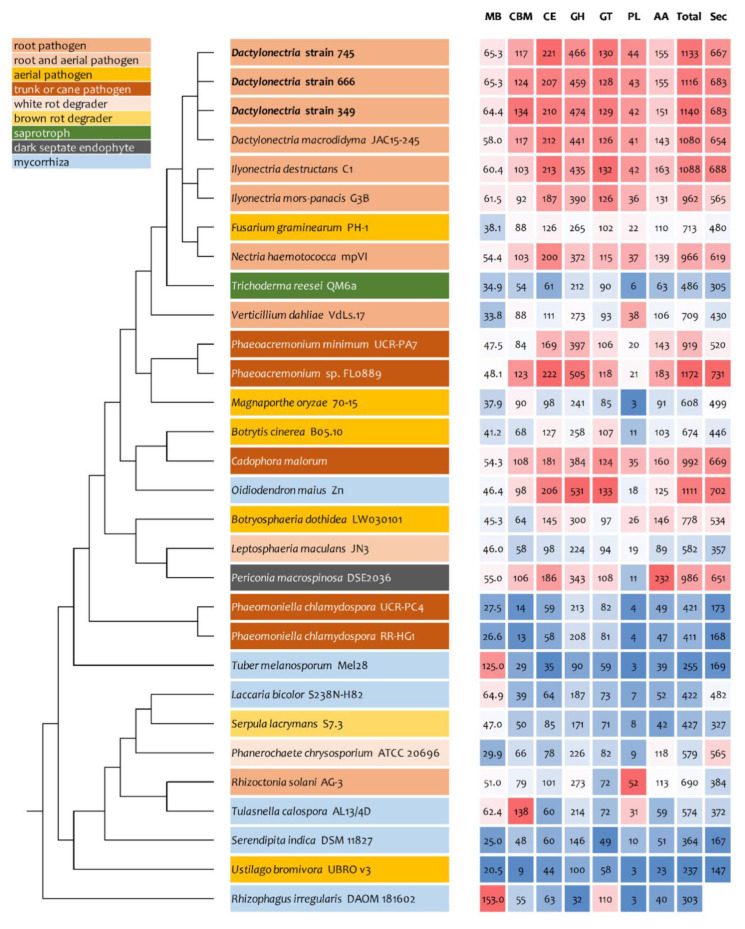
Comparative analyses of genome size, numbers of genes related to carbohydrate-active enzymes (CAZyme) and secretome in *Dactylonectria* *torresensis* strains BV-349, BV-666, BV-745 to known fungal pathogens, saprotrophs or symbionts. CBM: Carbohydrate-Binding Module, CE: Carbohydrate Esterase, GH: Glycoside Hydrolase, GT: Glycosyl Transferase, PL: Polysaccharide lyases, AA: Auxiliary Activity, Total: total number of CAZymes, Sec: number of secreted proteins.

As only small differences were recorded between the strains for either genes related to glycoside hydrolases (Figure 2), auxiliary activities and polysaccharide lyases (Figure 3), carbohydrate-binding modules, carbohydrate esterases (Figure 4) or glycosyl transferases (Figure 5), more information were searched in relation/differences of *Dactylonectria* strains to other fungi. Circos simulation shows relationship between the percentages of CAZymes genes shared by *Dactylonectria* strains and also with other black-foot fungi (Supplementary File 2: Figure S2). CAZyme numbers appears as significantly higher in *Dactylonectria* spp. than the average for the other 27 fungal genomes (Figure 6), which agrees with characteristics of plant pathogens [66].

In comparison to *Dactylonectria* strains described in this study, the dark septate endophyte *Oidiodendron maius* Zn has a total of 1111 genes related to CAZymes and the trunk disease pathogen *Phaeoacremonium* sp. FL0889 1172 (Figure 6). The highest score was also related to black-foot pathogens such as *Dactylonectria macrodidyma* JAC15-245 (1080 genes), and *Ilyonectria destructans* C1 (1088). Other genomes had genes numbers between 237 and 992.

The fact that these three genomes possess a high number of CAZymes and PCWDE domains suggests that the strains have a broad spectrum of enzymes as weapons for degrading plant cell-wall and to establish themselves as endophytes inside plant tissues as well as to macerate root tissues. Several genomic studies have indeed indicated that a high amount of CAZyme and PCWDE-related genes is linked to non-pathogenic or pathogenic endophytes [43,52,67].

### 3.5. Genes Involved in Oxidative Degradation of Plant Biomass

CAZyme gene analysis revealed the characteristic expansions in gene families implicated in infection and pathogenicity [39,66]. Here, in particular the increased number of GH43 encoding genes, but also of auxiliary functions suggest a role of the competence to degrade lignocellulose in pathogenicity of *Dactylonectria* spp. Therefore, we investigated the genomic content for the presence of AA3-1, AA9 and AA14 members. The AA9 family represents an efficient group of lytic polysaccharide monooxygenases (LPMOs) of high biotechnological relevance [68] due to their contribution to cellulose degradation, but also xyloglucan degradation was shown for this family [69]. A contribution of LPMOs to lignin degradation was reported as well [70] For AA14 LPMOs, only recently a high efficiency in boosting wood saccharification was shown [71]. LPMOs degrade lignocellulosic biomass via an oxidative mechanism, that requires an electron donor [72]. Cellulose dehydrogenases (CDHs) of the family AA3-1 are known to reduce LPMOs in nature and are hence crucial for their function [73]. Hence, the combination of these LPMOs and CDHs along with the fact that LPMOs were also found to be efficient under anaerobic conditions [74], make them ideal candidates for virulence factors of *Dactylonectria*.

For AA14, a Blastp search of the 321 Sordariomycetes genomes in Mycocosm (https://mycocosm.jgi.doe.gov) with the characterized *Pyconoporus coccineus* AA14 proteins [71] revealed putative homologues (e-values around 1E-38) in only about half of the Sordariomycetes species so far sequenced. In only a few cases, two putative homologues were detected. In the three *Dactylonectria* strains investigated here, we found one putative homologue each, but with amino acid identities around 25% and similarities around 31% to *P. coccineus* AA14a and AA14b. Since for those proteins no functional domains were characterized yet, a comparable functionality of the *Dactylonectria* homologues remains to be confirmed.

Members of the class AA9 (formerly GH61) are particularly important for oxidative degradation of plant biomass. *Dactylonectria* strains BV-666 and BV-745 contain twelve homologues with the respective domain, while BV-349 only has ten putative LPMOs of class AA9 (Supplementary File 3: Figure S3).

Analysis of putative cellobiose dehydrogenases, which fuel LPMO efficiency, was done in comparison of characterized homologues from www.cazy.org. A strikingly high number of putative homologues was detected in the *Dactylonectria* strains, with four in BV-349 and BV-666 and even five in BV-745, while for example *N. crassa* contains 2 and *T. reesei* none. However, their functionality as cellobiose dehydrogenases remains to be confirmed.

In summary, while the functionality of the additional homologues remains to be experimentally confirmed along with their expression in vivo, the high number of putative CDH and AA9 encoding genes supports the role of *Dactylonectria* as a pathogen with an efficient machinery for wood degradation. Despite important possible contributions of other factors to virulence, a clear difference for the three strains according to their isolation site (soil, grapevine, weed) could not be deduced from abundance of genes predominantly associated with wood degradation.

### 3.6. Analyses of Secretome and Small Secreted Proteins (SSP)

**Figure 7:**
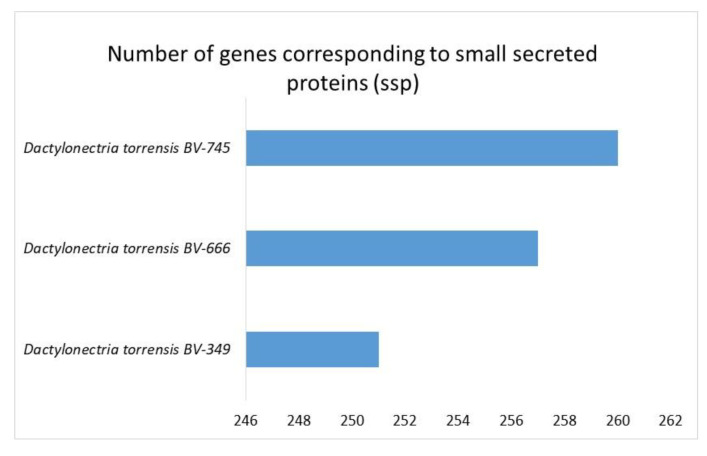
Numbers of genes in *Dactylonectria* *torresensis* strains BV-349, BV-666, BV-745 corresponding to small secreted proteins (ssp).

High numbers of genes of *D. torresensis* corresponding to secretome and a larger size of small secreted proteins were further characterized in the three genomes of *Dactylonectria* (with number between 683 and 687 secreted proteins and 251–260 for SSP) (Figure 6 and Figure 7). Comparison showed that dark septate endophytes *Pericornia macrospinosa* DSE2036 and *Oidiodendron maius* Zn as well as trunk disease pathogen *Cadophora malorum*, *Phaeoacremonium* sp. FL0889, and black-foot pathogens *Dactylonectria macrodidyma* JAC15-245, as well as *Ilyonectria destructans* C1 had a similar range of genes related to secretome (650–731) (Figure 6). Small secreted proteins play important roles in pathogenicity of fungal-plant interactions and in symbiosis [52,75,76,77]. The large numbers of secreted proteins in *Dactylonectria* strains show also potential roles in pathogenicity.

### 3.7. Antismash Analyses

One of the crucial weapons for fungal plant pathogen is the production of phytotoxic compounds [78]. Fungal antismash analysis was used to search in the genomes’ gene clusters encoding key enzymes such as NRPS (non-ribosomal peptide synthetase), PKS (polyketide synthase), HYBRID PKS-NRPS and others. *Dactylonectria* strains were found to contain a significant number of genes encoding key secondary metabolism biosynthesis enzymes (Table 4). Interestingly, biosynthetic gene clusters for echinocandin B (antifungal lipopeptide inhibiting the synthesis of glucan), brefeldin (antiviral metabolite) and asperfuranone (a polyketide) were detected in the *Dactylonectria* genomes, except in BV-349 for echinocandin B (Table 4). Surprisingly, a fujikurin biosynthetic gene cluster was also detected in all three genomes with percentage of similarity between 83 and 100% to known gene cluster (Table 4). Fujikurin has been isolated from an endophytic *Fusarium* species [79] and phytopathogens and highlights were made on a possible role of this metabolite as a phytopathogenic virulence determinant [80]. Other fujikurin-like clusters were also detected in opportunistic pathogens such as *Aureobasidium pullulans* and *Scedosporium* spp., in the saprophyte *Endocalyx cinctus* (saprophyte in dead palms), in the ericoid mycorrhizal fungus *Cairneyella variabilis*, in *Paecilomyces hepiali* (entomopathogenic fungus associated with plants) and *Ophiostoma* sp. responsible of Dutch elm disease [81]. The presence of this gene cluster, but with a configuration different from the one of *Fusarium fujikuroi*, a species complex belonging to *Nectriaceae* [82] (Supplementary File 4: Figure S4) suggests that this secondary metabolite could allow several fungal species to interact with plants, as phytopathogens or non-pathogenic endophytes.

### 3.8. Light Response

**Figure 8:**
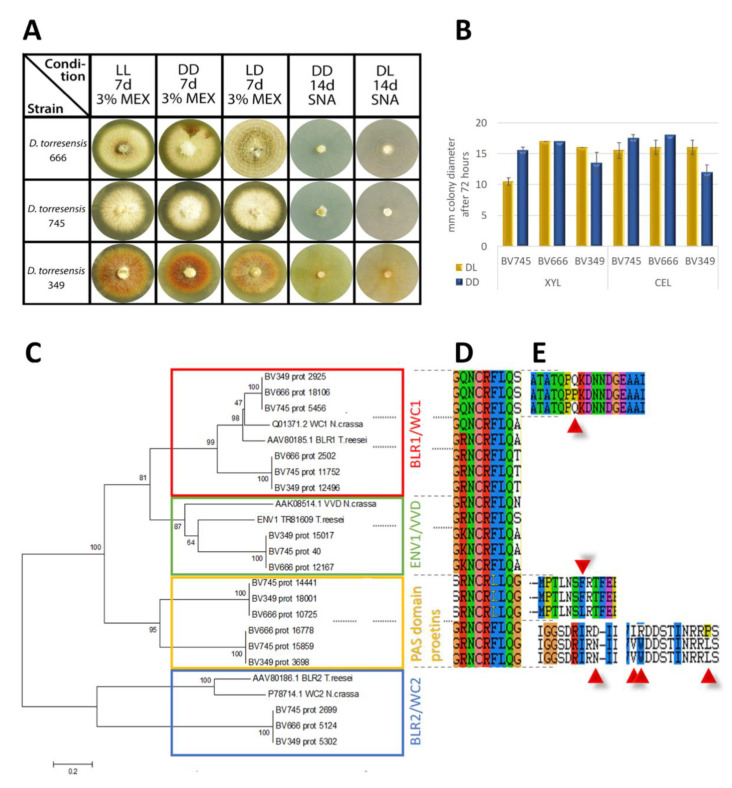
Light response and photoreceptors in *Dactylonectria*. (**A**) Growth characteristics of mycelia grown on 3% (*w*/*v*) malt extract (MEX) or SNA medium in daylight (DL; 12:12 dark:light cycles) or constant darkness (DD) for 7 days. For strain BV-666 formation of rings is visible on both media. (**B**) Analysis of hyphal extension upon growth on Mandels Andreotti minimal medium (Mandels 1978) with 1% (*w*/*v*) xylan or cellulose in daylight (DL) or constant darkness (DD). Errorbars reflect standard deviation of two biological replicates. (**C**) Phylogenetic analysis of photoreceptor homologues in *Dactylonectria*. The evolutionary history was inferred using the minimum evolution method (Eck and Dayhoff 1966). The bootstrap consensus tree inferred from 500 replicates is taken to represent the evolutionary history of the taxa analyzed (Felsenstein 1985). Phylogenetic analyses were conducted in MEGA4 (Tamura et al. 2007). GenBank accession numbers of reference proteins are given with the protein names and species. (**D**) Encoded amino acids in the genomic region of the NCRFLQ conserved motif in the proteins shown in the phylogenetic tree. (**E**) Alterations in the amino acid sequence at different loci of proteins shown in the phylogenetic tree.

Light has a profound impact on physiology and metabolism of fungi [83] and in particular also on regulation of plant cell-wall degradation [84,85], which may be relevant for pathogenicity of *Dactylonectria*. Light and photoreceptors impact circadian rhythmicity as well and recently a connection of fungal circadian rhythmicity of pathogenicity was detected [86,87]. We noticed that the three strains investigated in this study show phenotypic differences when grown in light (Figure 8A). Especially, BV-666 showed a clear reaction to changing light conditions when it was grown in daylight (light:dark 12 h:12 h). Differences in hyphal extension between daylight and darkness were obvious on xylan (BV-745) or cellulose (BV-349) (Figure 8B). Therefore, we were interested if these differences are reflected in the genomes of these strains. We analyzed blue light photoreceptor candidates of all three *Dactylonetria* strains and found that the photoresponse machinery of these fungi is more complex than that of *N. crassa* or *T. reesei*. Besides homologues of ENV1/VVD and BLR2/WC-2, *Dactylonectria* spp. have an additional, close homologue to BLR1/WC-1, representing the crucial PAS/LOV photoreceptors. Moreover, we found two further proteins related to and sharing similar domains with BLR1/WC1 (Figure 8C). All *Dactylonectria* proteins containing a putative PAS/LOV (Per-ARNT-Sim/Light, oxygen or voltage) domain also comprised the conserved sequence NCRFLQ which is considered crucial for light responses [88], except for one of them (Figure 8D), where the sequence is altered to NCRLLQ. Then, we tested whether alterations in the sequences of the detected photoreceptors would correlate with the altered light response we had observed. Indeed, aligning the sequences revealed that in BV-666, three of the four photoreceptor homologues showed alterations in one or more amino acids (Figure 8E), which is in accordance with an altered response to daylight (Figure 8A). However, since these alterations did not occur in functional domains like phosphorylation or myristoylation sites, we could not assign a possible relevance. Since BV-666 was isolated from grapevine, further work to evaluate whether the detected alterations may have affected association with the plant and hence virulence, could provide insight into the relevance of the extended photoresponse system of *Dactylonectria* on pathogenicity.

The sequence differences in the photoreceptors described above may impact circadian rhythms already, but in addition we analyzed the sequence of FREQUENCY (FRQ), which is crucial for circadian rhythmicity in *N. crassa* [89]. However, we did not detect any differences in FRQ between the strains.

## 4. Conclusions

In this study, we analyzed genomes of *D. torresensis* strains from three different habitats such as soil, weed, and grapevine. Overall, there was a similar genome content in the strains with genes related to necrosis, heavy metal/metalloid resistance, salicylic acid degradation, and a high number of CAZymes related to glycoside hydrolases that could be involved in non-pathogenic endophytism or pathogenicity. Analysis of photoreceptors revealed an increased number as well as specific mutations in one strain, indicating an increase relevance of light perception and potentially integration with other signals in *Dactylonectria* compared to other ascomycetes. High numbers of genes related to secretome and small secreted proteins were further detected. However, small differences were recorded between the three genomes. Our analysis also demonstrates the presence of several gene clusters with some as fujikurin-like genes that have been linked to fungal pathogenicity. We further detected high numbers of genes related to CAZyme families and high numbers of transposons. This finding is further represented by CAZymes associated with wood degradation.

The fact that *D. torresensis* can be a non-pathogenic endophyte on weeds or grapevine has important implications. The potential role of asymptomatic hosts may include not only preservation of a viable inoculum source quantitatively during for example, crop rotation in grapevine nursery fields, but also in shaping the genetic structure of the pathogen population qualitatively, which may have significant implications for disease management. This finding also highlights the urgent need to implement early, accurate and specific *in planta* detection and quantification of *D. torresensis* to prevent the spread of black-foot disease in grapevine propagation material. The future direction of research on black-foot needs to investigate: (i) how these fungi colonize roots of secondary hosts or grapevine and establish themselves inside, and (ii) what triggers latent black-foot fungi to transition from a non-pathogenic endophyte to a pathogenic endophyte, and cause disease symptoms in grapevine.

## Figures and Tables

**Table 1:** Genome statistics of *Dactylonectria* strains.

		Strain	
Genome Statistics	BV-349	BV-666	BV-745
Genome assembly size (Mbp)	64.42	65.33	64.21
Number of reads	19,695,918	32,400,268	25,108,162
Mapped reads	98.23%	97.44%	98.65%
Unmapped reads	1.77%	2.56%	1.35%
Coverage	42.23×	67.88×	53.58×
Completeness	98.6%	98.3%	99%
Duplication ratio	1.008	1.008	1.007
Largest alignment	242,734	329,511	254,604
Total aligned length	48,129,906	48,518,112	48,295,324
Contigs	2809	2598	2396
Largest contig	377,758	402,120	458,724
GC (%)	50.67	50.17	50.23
Proteins encoding genes	19,102	19,090	18,724

**Table 2:** Clusters of ortholog groups of *Dactylonectria* strains.

COG Categories	BV-349	BV-666	BV-745
RNA processing and modification [A]	342	321	336
Chromatin structure and dynamics [B]	125	121	131
Energy production and conversion [C]	657	671	665
Cell cycle control, cell division, chromosome partitioning [D]	370	347	329
Amino acid transport and metabolism [E]	774	790	780
Nucleotide transport and metabolism [F]	207	191	186
Carbohydrate transport and metabolism [G]	1287	1321	1300
Coenzyme transport and metabolism [H]	213	222	216
Lipid transport and metabolism [I]	548	542	536
Translation, ribosomal structure and biogenesis [J]	412	419	415
Transcription [K]	536	558	541
Replication, recombination and repair [L]	424	432	439
Cell wall/membrane/envelope biogenesis [M]	399	356	345
Cell Motility [N]	3	3	3
Posttranslational modification, protein turnover, chaperones [O]	729	736	702
Inorganic ion transport and metabolism [P]	359	369	357
Secondary metabolites biosynthesis, transport and catabolism [Q]	1018	1062	1020
General function prediction only [R]	0	0	0
Function unknown [S]	7790	7853	7720
Signal transduction mechanisms [T]	669	630	595
Intracellular trafficking, secretion, and vesicular transport [U]	427	415	423
Defense mechanisms [V]	113	123	117
Extracellular structures [W]	8	9	8
Nuclear structure [X]	28	24	24
Cytoskeleton [Y]	194	177	189
TOTAL	17,632	17,692	17,377

**Table 3:** Genes related to pathogenicity, metal resistance or other properties in *Dactylonectria* genomes.

Clusters of Genes Related to Pathogenicity		Strain	
Metal Resistance or Other Properties	BV-349	BV-666	BV-745
**Genes related to necrosis**	11	10	10
Necrosis and ethylene inducing peptide	2	2	2
Necrosis inducing protein (NPP1)	9	8	8
**Genes related to carbohydrate-actives enzymes**	1140	1116	1133
Carbohydrate-binding modules	134	124	117
Carbohydrate esterases	210	207	221
Glycoside hydrolases	474	459	466
Glycosyl transferases	129	128	130
Polysaccharide lyases	42	43	44
Auxiliary activities	151	155	155
**Genes related to heavy metal/metalloid resistance**	48	37	36
Copper	22	23	23
Cadmium	2	5	2
Other heavy metal/metalloids	8	5	3
**Genes related to other properties**	23	18	22
Salicylate hydroxylase	18	13	17
Siderophore	4	4	4
Auxin	1	1	1

**Table 4:** Antismach analysis of *Dactylonectria* genomes.

		Strain	
Antismash Analysis	BV-349	BV-666	BV-745
Terpene (number of gene clusters)	8	9	9
T1pks (number of gene clusters)	15	17	16
Nrps (number of gene clusters)	7	10	9
T1pks-nrps (number of gene clusters)	2	2	2
T3pks (number of gene clusters)	1	1	1
Other (number of gene clusters)	7	7	8
Brefeldin_biosynthetic gene cluster (% similarity)	50	20	13
Echinocandin_B_biosynthetic gene cluster (% similarity)	-	13	40
Asperfuranone_biosynthetic gene cluster (% similarity)	27	27	27
Brefeldin_biosynthetic gene cluster (% similarity)	-	40	20
Fujikurins_biosynthetic gene cluster (% similarity)	83	100	83

## Data Availability

This Whole Genome Shotgun (WGS) Project has been deposited at DDBJ/ENA/GenBank under the accessions VYKH00000000 (*D. torresensis* isolate BV-349), VYKG00000000 (isolate BV-666) and VYKF00000000 (isolate BV-745). The versions described in this paper are version VYKH01000000, VYKG01000000 and VYKF01000000, respectively. This *Dactylonectria torresensis* Genome Sequencing and Assembly Project was submitted under BioProject PRJNA566152.

## Supplementary Materials

The following are available online at https://www.mdpi.com/2309-608X/6/4/255/s1, Figure S1: Average Nucleotide Identity (ANI). Figure S2: circular network plot of CAZymes categories and their percentages shared in *Dactylonectria torresensis* strains (top) and with other black-foot pathogens (below). A: CBM, B: CE, C: GH, D: GT, E: PL, F: AA, G: BV-745, H: BV-666, I: BV-349, J: *Ilyonectria destructans* C1, K: *Ilyonectria mors-panacis* G3B, L: *Dactylonectria macrodidyma* JAC15-245. Figure S3: phylogenetic analysis of AA9 candidates of *Dactylonectria*. The evolutionary history was inferred using the maximum parsimony method (Eck and Dayhoff 1966). The bootstrap consensus tree inferred from 500 replicates is taken to represent the evolutionary history of the taxa analyzed (Felsenstein 1985). Phylogenetic analyses were conducted in MEGA4 (Tamura et al., 2007). GenBank accession numbers of reference proteins are given with the protein names and species. Figure S4: antismash analyses of fujikurin-like genes in *Dactylonectria torresensis* strains BV-349, BV-666, BV-745.

## Abbreviations

Average Nucleotide Identity (ANI); Carbohydrate-Active Enzymes (CAZymes); Carbohydrate Esterases (CE); Carbohydrate-Binding Modules (CBM); Fusarium Head Blight (FHB); Glucose-Faba Bean Rose Bengal Agar (GFBRBA); Glycoside Hydrolases (GH); Glycoside Transferases (GT); Hasegawa-Kishino-Yano model (HKY); Internal Transcribed Spacer (ITS); Malt Extract Agar (MEA); Polysaccharide Lyases (PL); and Potato Dextrose Agar (PDA).
