# Supplementary material for: Comparative Genomic Analysis of Dactylonectria torresensis Strains from Grapevine, Soil and Weed Highlights Potential Mechanisms in Pathogenicity and Endophytic Lifestyle
Source: J Fungi (Basel). 2020 Oct 29;6(4):255. doi: 10.3390/jof6040255 (PMC7712071; doi:10.3390/jof6040255)
Supplement: Supplementary file 1 [file jof-06-00255-s001.zip › jof-965959-supplementary.pptx]

## Slide 1
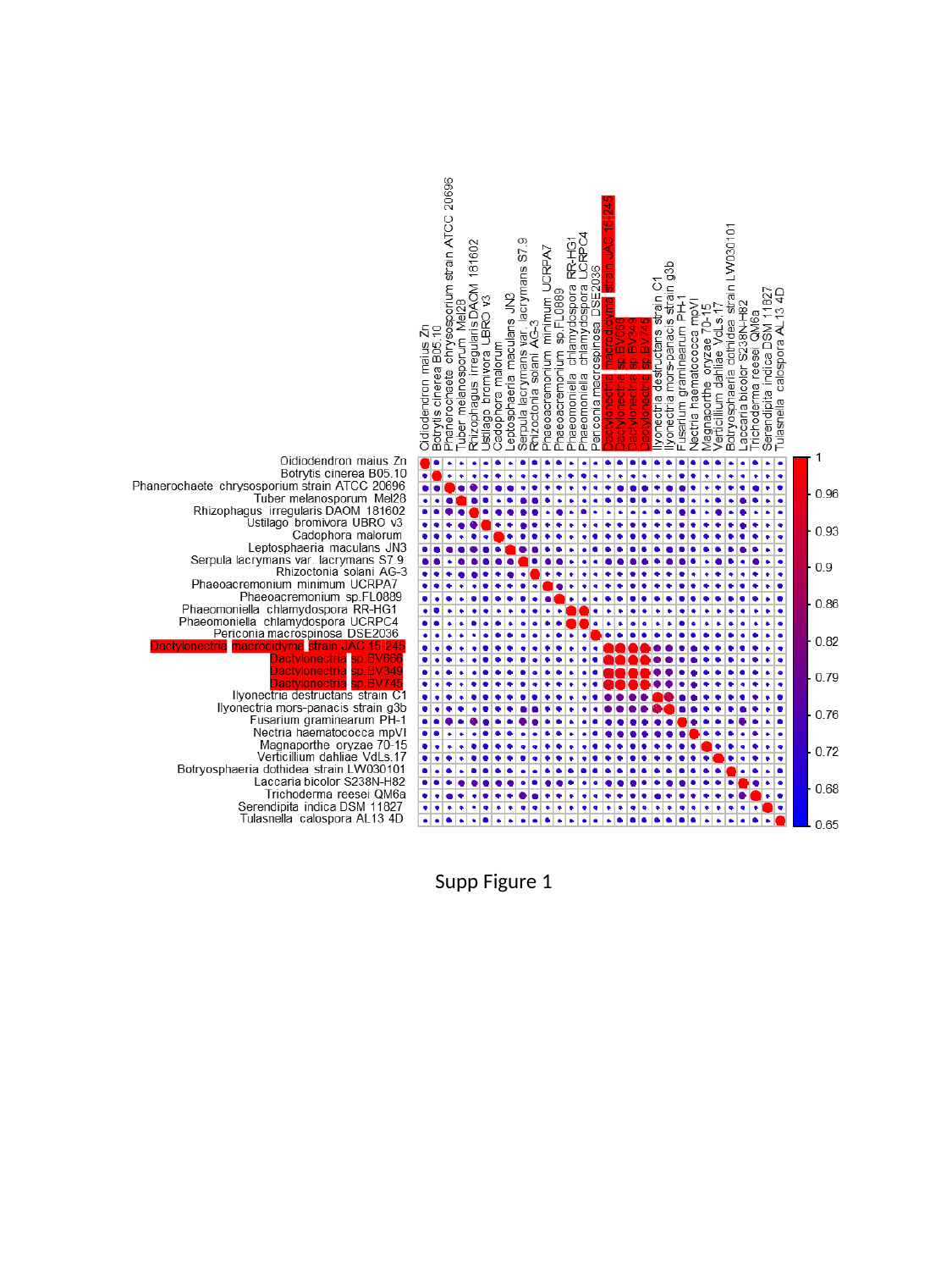

Supp Figure 1

## Slide 2
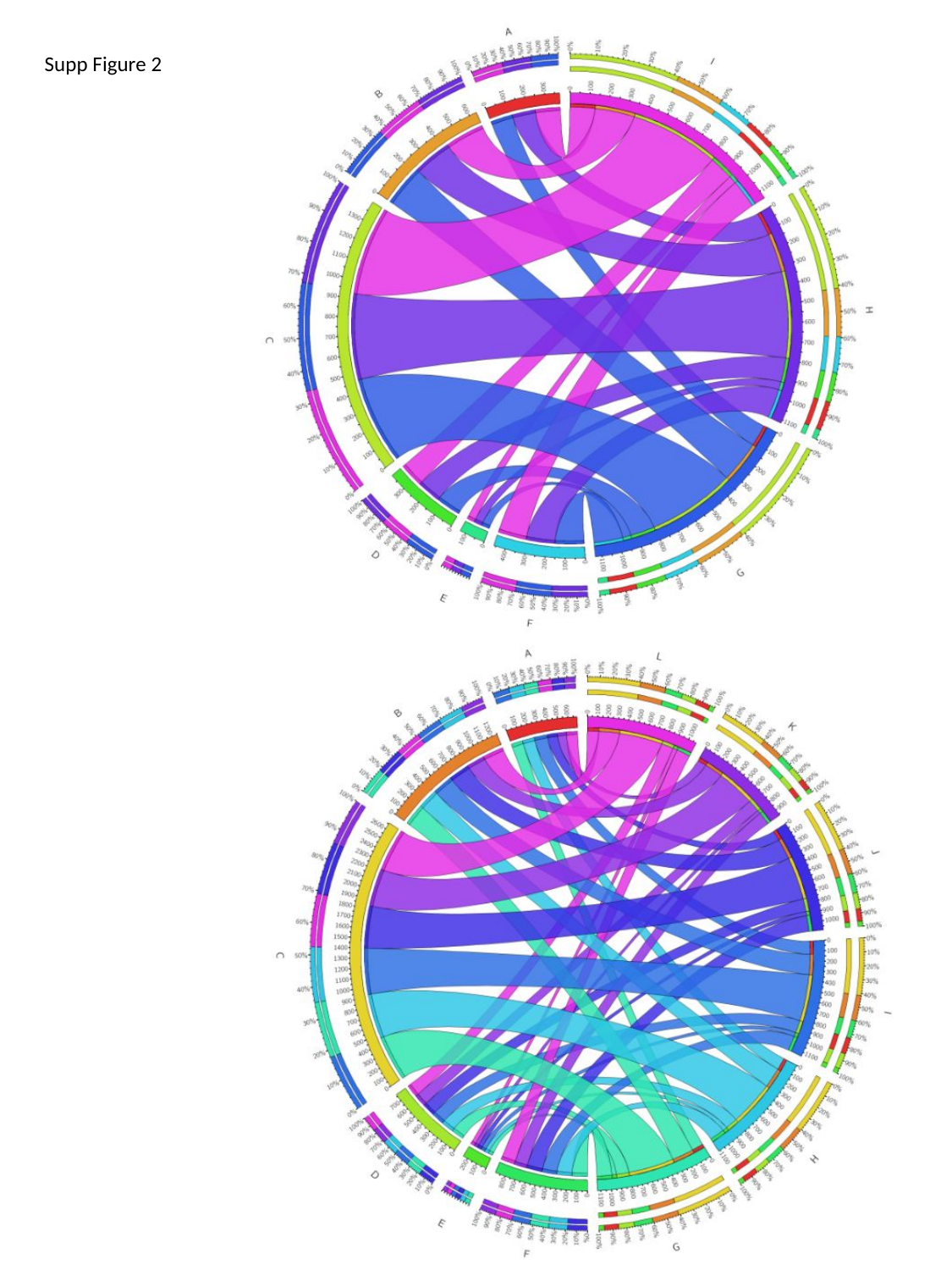

Supp Figure 2

## Slide 3
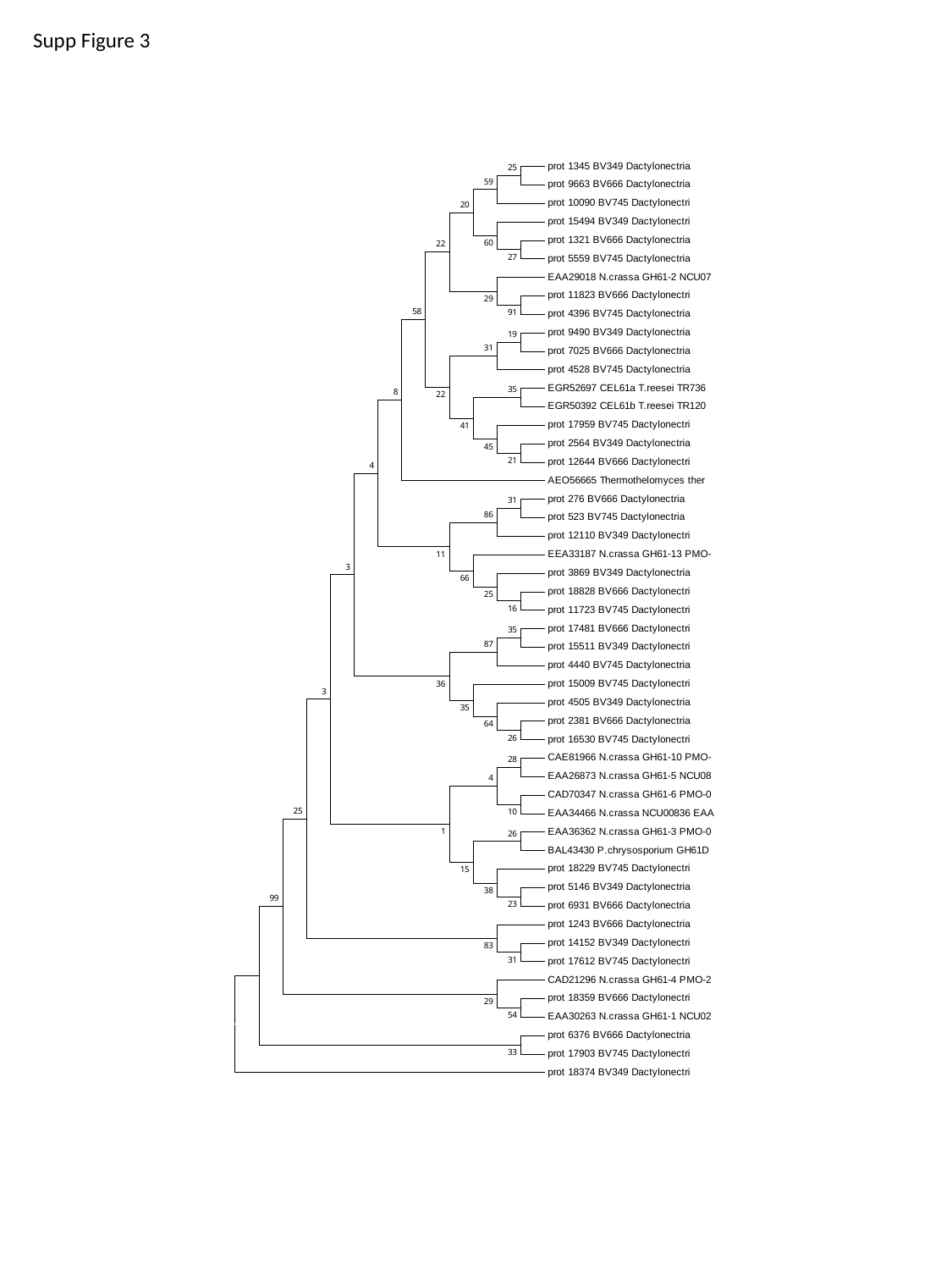

Supp Figure 3

## Slide 4
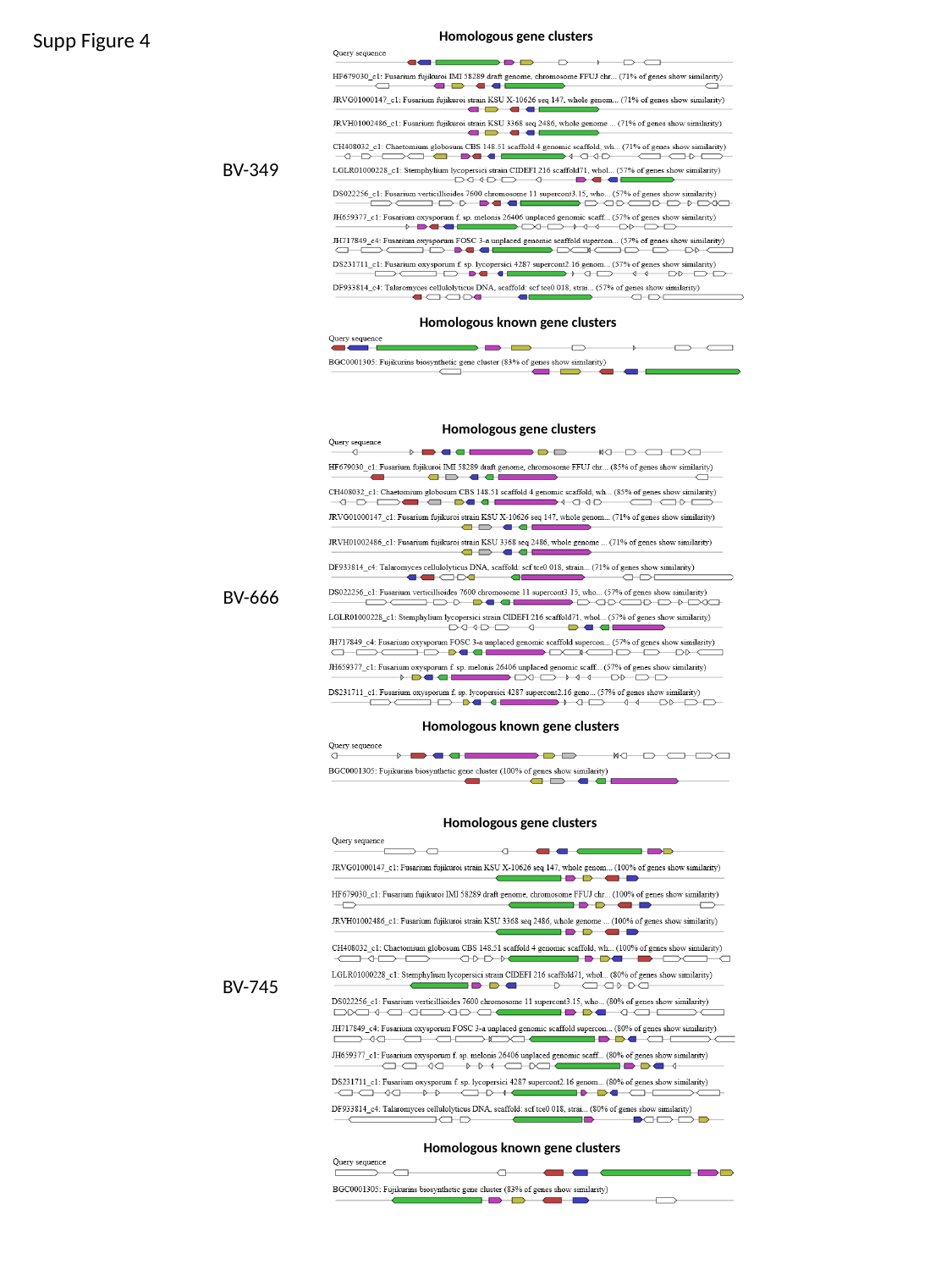

Supp Figure 4
Homologous gene clusters
BV-349
Homologous known gene clusters
Homologous gene clusters
BV-666
Homologous known gene clusters
Homologous gene clusters
BV-745
Homologous known gene clusters
